# An assessment of physician assistant student diversity in the United States: a snapshot for the healthcare workforce

**DOI:** 10.1186/s12909-022-03717-9

**Published:** 2022-09-15

**Authors:** Carolyn Bradley-Guidry, Nicole Burwell, Ramona Dorough, Vanessa Bester, Gerald Kayingo, Sumihiro Suzuki

**Affiliations:** 1 Department of Physician Assistant Studies, School of Health Professions, University of Texas Southwestern Medical Center, Dallas, TX USA; 2 Stanford School of Medicine, Stanford, CA USA; 3 University of Texas Southwestern Medical Center, Dallas, TX USA; 4 Augsburg University, Minneapolis, MN USA; 5 Physician Assistant Leadership and Learning Academy, Graduate School, University of Maryland Baltimore, Baltimore, MD USA; 6 Section of Biostatistics and Epidemiology, Department of Family and Preventive Medicine, Rush University Medical Center, Chicago, IL USA

**Keywords:** Diversity, PA students, Graduates, Top-performing PA programs, Physician Assistants

## Abstract

**Background:**

The Physician Assistant (PA) workforce falls short of mirroring national demographics mainly due to a lack of diversity in student enrollment. Few studies have systematically examined diversity across PA programs at the national level, and little is known about best practices for consistently graduating a diverse group of students. We descriptively characterized the extent to which PA programs are graduating a diverse group of students and identified top performing PA programs.

**Methods:**

Data from the Integrated Postsecondary Education Data System (IPEDS) were used to calculate the number and proportion of racial or ethnically diverse graduates. The study sample included 139 accredited PA programs that had graduated a minimum of five cohorts from 2014–2018. Within each of the United States Census Divisions, programs were ranked according to the number and proportion of graduates who were underrepresented minority (URM) race, Hispanic ethnicity, and of non-white (URM race, Hispanic, and Asian).

**Results:**

Amongst PA programs in the United States, a large disparity in the number and proportion of racial and ethnic graduates was observed. Of 34,625 PA graduates, only 2,207 (6.4%) were Hispanic ethnicity and 1,220 (3.5%) were URM race. Furthermore, a large number of diverse graduates came from a small number of top performing programs.

**Conclusion:**

Despite the abundance of evidence for the need to diversify the healthcare workforce, PA programs have had difficulty recruiting and graduating a diverse group of students. This study provides empirical evidence that PA programs have not been able to attain the level of diversity necessary to shift the lack of diversity in the PA workforce. Based upon this study's findings, the top performing PA programs can be used as role models to establish benchmarks for other programs. The results of this descriptive study are currently being used to guide a qualitative study to identify the top performers’ strategies for success.

**Supplementary Information:**

The online version contains supplementary material available at 10.1186/s12909-022-03717-9.

## Background

The Physician Assistant (PA) profession has undergone phenomenal growth in the past 50 years; nonetheless areas of diversity, inclusion, and health care equity remain a challenge [1–3]. The percentage of minority PAs remains disproportionately small despite many efforts to increase workforce diversity over the past several decades [4–7]. The National Commission on Certification of Physician Assistants (NCCPA) reported that over 80% of practicing PAs were white from 2016–2020 [8]. In the same five-year period, the number of certified PAs identifying as Black/African American declined from 3.6% (2016) to 3.3% (2020). Increasing racial and ethnic diversity is crucial to achieving a PA workforce with the capacity to provide accessible, equitable, and culturally competent health care to the nation’s changing demographic population [9].

A significant barrier to a diverse PA workforce is the lack of diversity amongst matriculating students to PA education programs. There is substantial evidence in the literature supporting the importance and the need for diversity in PA and other health profession education [10–16]. The United States (U.S.) Supreme Court has long recognized and acknowledged that student diversity is of compelling interest for educational program admission practices. According to the U.S. Department of Education, diverse learning environments sharpens critical thinking and analytic skills [17]. Despite these facts, the Physician Assistant Education Association (PAEA) reports that 69.4% of first-year PA students were white, 7.6% Hispanic and 3.9% Black [18]. The Central Application Service for Physician Assistants (CASPA) show that underrepresented groups continue to matriculate at much lower rates [19, 20]. If the PA profession were to continue to train new PAs at current diversity proportions, the current training and supply of future PAs will do very little to diversify the workforce and does not correspond to the growing demographic shift of the U.S. population [2, 5, 21–23].

In its strategic plan, the PAEA aspires to promote diversity in all aspects of PA education. It recommends that programs should recruit a diverse faculty and student body [24–26]. The American Academy of Physician Assistants (AAPA) has identified equity as one of the core values through which it seeks to eliminate disparities and barriers to quality health care. The AAPA has also identified promotion of inclusion as one of the strategic commitments to the profession between 2016–2020 [27]. The 5th edition of the Accreditation Review Commission on Education for the Physician Assistant (ARC-PA) standard (A1.11) states “the sponsoring institution must demonstrate its commitment to student, faculty and staff diversity and inclusion by:

- a) supporting the program in defining its goal(s) for diversity and inclusion,
- b) supporting the program in implementing recruitment strategies,
- c) supporting the program in implementing retention strategies, and
- d) making available, resources which promote diversity and inclusion” [28].

The purpose of the standard is to guide PA programs in developing and implementing strategies to foster diversity and inclusion of students, faculty, and staff in PA education programs. Despite these efforts, diversifying the student body and resulting workforce has been a challenge. To our knowledge, only a few studies have systematically examined diversity in PA programs at the national level [10]. There are no published studies that have longitudinally examined the PA workforce using national data to identify consistent contributors to diversity. Further, no studies have provided evidence on “what works”, or benchmarks for success to achieve diversity in PA programs. To this end, we conducted a secondary data analysis study, to quantitatively identify top performing programs as the first step toward establishing PA diversity benchmarks and best practices. This paper presents the quantitative descriptive study that identifies the top performing PA programs per U.S. Census Division that have contributed to racial and ethnically diverse PA graduates from 2014–2018.

## Methods

### Data source

The institutional review board at the University of Texas Southwestern Medical Center reviewed and deemed the mixed-methods quantitative-dominant, complementary theoretical drive research design as being in the exempt category [29]. This part of the study utilized quantitative analysis of data from the Integrated Postsecondary Education Data System (IPEDS) [30]. All data analyses were conducted using SPSS version 26 (IBM Corp, Armonk, NY). IPEDS data were obtained from annual surveys conducted by the U.S. Department of Education’s National Center for Education Statistics [30]. All U.S. postsecondary institutions that participate in federal financial aid programs are required to complete the surveys. IPEDS data contain, among others, completions data that include the number of students who completed a postsecondary education program by type of program and level of award [30]. PA programs were identified using the IPEDS Classification of Instructional Programs (CIP) code (CIP code 51.0912). The CIP code provides a taxonomic scheme that supports the accurate tracking and reporting of field of study and program completion [31]. Data were stratified by gender, race, and ethnicity allowing for programmatic comparisons of graduates. Inclusion and exclusion criteria were developed with the goal of including programs that best illustrate how PA programs have contributed to diversifying the workforce consistently over time.

Inclusion criteria: We included data from 139 accredited PA programs that graduated five consecutive cohorts of PA students from 2014 to 2018, with PA CIP code 51.0912. The goal of the inclusion criteria was to ensure all PA programs studied had 5 years of graduate data. The ARC-PA website was assessed in 2019, revealing 250 PA programs with accreditation status listed as continued, provisional, or probation [28]. We included accredited PA programs listed as continued or probation status.

Exclusion criteria: A total of 72 programs were excluded due to provisional status due to a lack of 5 years of graduate data. Another 21 programs were excluded based upon lack of an official NCCPA PANCE five-year, first-time taker summary report from 2014–2018. There were 157 PA programs that advanced to IPEDS review; 10 programs did not have a CIP code and 8 programs did not have documented IPEDS graduate data from 2014–2018 and were also excluded.

A total of 139 accredited PA programs with complete data of five consecutive cohorts of PA students from 2014 to 2018 were included in this study. Data were stratified by U.S. Census Divisions. This method was selected based on PAEA divisional reporting methods. Since 1984, PAEA has published PA programs and first-year student demographic data from the Program’s Survey [32]. The By the Numbers: Program Report identifies the number of PA programs located within a U.S. Census Division.

**Fig. 1:**
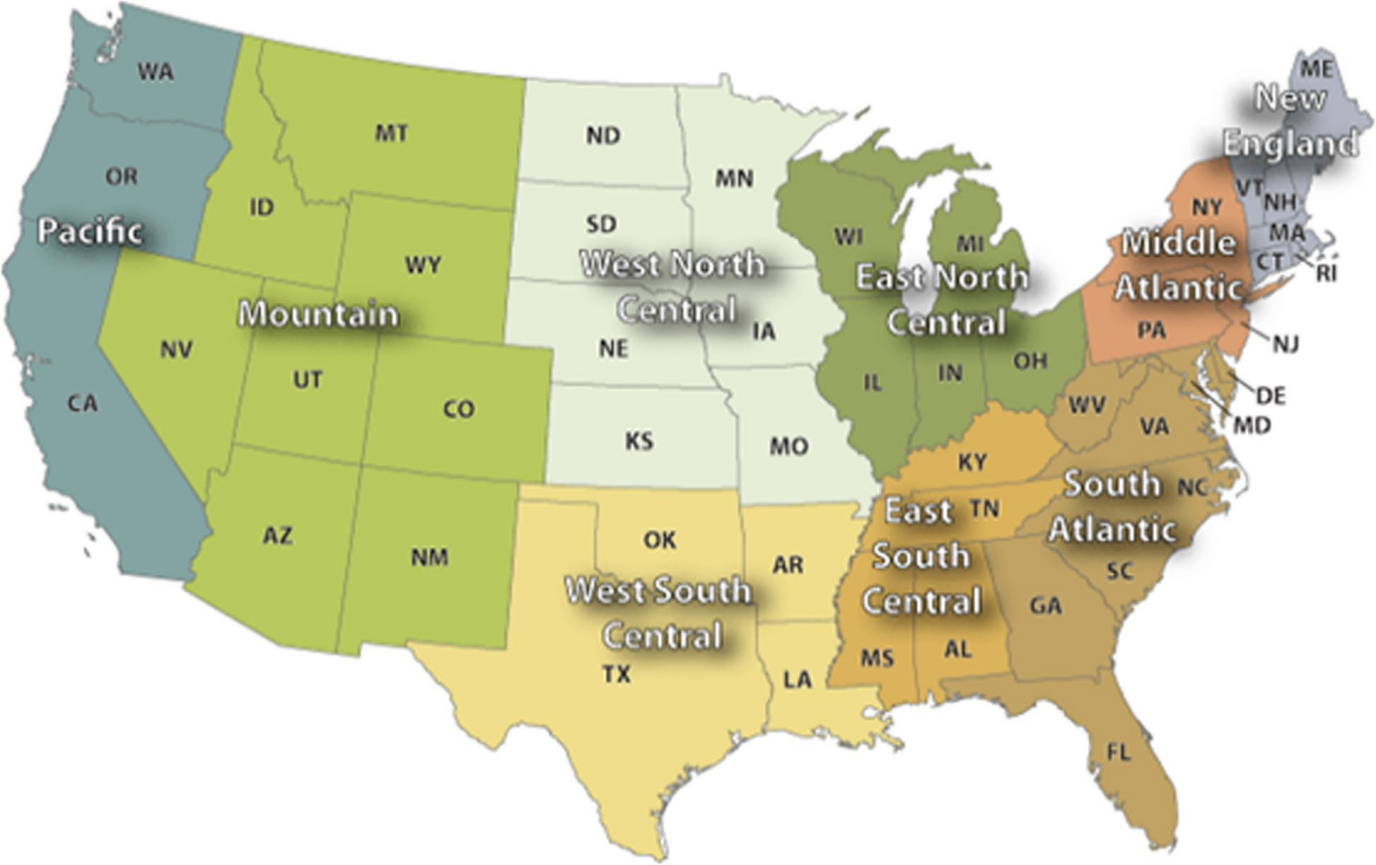
U.S. Census Divisions Map

To ensure consistency with PAEA reports, data were stratified by the following U.S. Census Divisions. 1. New England, 2. Middle Atlantic, 3. East North Central, 4. West North Central, 5. South Atlantic, 6. East South Central, 7. West South Central, 8. Mountain, and 9. Pacific [33, 34]. Figure 1 displays the U.S. Census Divisions [33].

### Definition of race and ethnicity

Race and ethnicity information in IPEDS is collected in two parts. Part one asks students their ethnicity by asking, “Are you Hispanic or Latino?” [35]. Part two asks students to choose one or more race from the following: American Indian or Alaskan Native, Asian, Black or African American, Native Hawaiian or Other Pacific Islander, or White. Students who answer “Yes” to the first question are categorized as “Hispanic” regardless of their response to the second question. Students who choose not to answer both questions as well as students who answer “No” to the first but do not answer the second are categorized into “Unknown race and ethnicity”. Students who do not answer the first but answer the second are assumed to be non-Hispanic and categorized into the corresponding race they selected. Anyone who selects more than one race is categorized into “Two or more races”. Thus, the race and ethnicity categories in the IPEDS data are mutually exclusive with no overlap. Students who are nonresident aliens do not answer these items and are categorized as “Nonresident alien” [35, 36]. Students categorized as unknown race/ethnicity or nonresident alien were excluded for this study.

### Top performing programs

There is no universal definition or measure of diversity within PA education. Therefore, we considered two simple yet informative measures: total number of diverse graduates and proportion of total graduates who are diverse. We chose to use both measures because they are equally important. While the total number of graduates indicates the actual number of racially/ethnically diverse PAs joining the workforce, the proportion (percent) indicates how well a program is doing in recruiting and graduating a diverse cohort of PA students.

We explored these two measures among three categories of diversity: non-white (URM race, Hispanic, and Asian), Hispanic/Latino ethnicity (Hispanic), and underrepresented minority (URM) race. The non-white category included graduates who were identified as Hispanic, American Indian or Alaskan Native, Asian, Black or African American, Native Hawaiian or Other Pacific Islander, or two or more races. We created the non-white group to include all students of color who contribute to the PA workforce diversity [37]. We chose not to separate Asian as racial category, because although Asians only make up 7% of the U.S. population, they tend to be overrepresented in higher education. However, excluding Asians altogether fails to capture the underrepresented subgroups with Asians, e.g., Hmong American. The IPEDS data do not contain information on these subgroups. The remaining two diversity categories captured ethnicity and races often considered to be underrepresented. Hispanic ethnicity included only those who were identified as Hispanic or Latino, and URM race included American Indian or Alaskan Native, Black or African American, and Native Hawaiian or Other Pacific Islander.

For each of the three categories, top performing programs were determined by calculating the number of diverse PA graduates over 5 years for each program. We also computed the proportion of graduates by dividing the sum of a program’s diverse graduates over 5 years by the total number of that program’s 5-year graduates. Within each of the nine U.S. Census Divisions, the “top performers” were defined as the top three programs with the highest total number as well as highest proportion of diverse graduates in each category. In cases of overlap, i.e., a program ranks in the top three in multiple categories, each category was separately ranked, and hence the program was counted as a top performer in every applicable category. In cases of ties, i.e., multiple programs’ ranks are the same and among the top three, all applicable programs were included.

## Results

The number of programs per Division ranged from a minimum of 8 programs (Divisions 6 and 9) to a maximum of 35 programs (Division 2) with a median of 10 programs. We identified 47 programs to be top performing programs based on the total number of graduates in the categories of non-white, URM race, or Hispanic. Similarly, we identified 45 programs to be top performing based on the proportion of graduates in the aforementioned categories. Overall, there were 61 unique programs identified as top performers in at least one category. Table 1 displays the 61 unique top performing PA programs. The complete list and rankings of all top performing programs is available as a Supplemental Appendix 1 and 2. The rankings are based on both numbers [Supplemental Appendix 1] and proportions [Supplemental Appendix 2] of PA graduates per division.

**Table 1:** Top PA program diversity performers based on both number and proportion of URM/non-white graduates per US Census Division from 214–2018 (*n* = 61)^c^

US Census Division	Top PA program diversity performers								
1 New England	^a^MA College of Pharmacy and HS^b^	University of Bridgeport	Quinnipiac University	Springfield College	Bay Path University	Franklin Pierce University			
2 Middle Atlantic	CUNY-City College	SUNY Downstate HS^b^ University	CUNY-York College	Touro College	St. John’s University New York	Pace University	Drexel University		
3 East North Central	Northwestern University	University of Toledo	Midwestern University Downers Grove	Rosalind Franklin University of Medicine	University of Detroit Mercy				
4 West North Central	University of Nebraska Medical Center	University of North Dakota	Wichita State University	Missouri State University-Springfield	Union College	University Iowa	Des Moines University-Osteopathic Medicine		
5 South Atlantic	Miami Dale College	Nova Southeastern University	South University- Tampa	Keiser University-Ft. Lauderdale	Emory University	Anne Arundel Community College	Eastern Virginia Medical School	Barry University	Duke University
6 East South Central	Bethel University (Tennessee)	Mississippi College	Lincoln MemorialUniversity	University of Alabama at Birmingham	South College	University of South Alabama			
7 West South Central	University of Texas South-western Medical Center	University of Texas Rio Grande Valley	University of Texas HS^b^ at San Antonio	Texas Tech University HS^b^ Center	Louisiana State University HS^b^ Center—Shreveport	Franciscan Missionaries of Our Lady University	University of Texas Medical Branch	University of North Texas HS^b^ Center	University of Oklahoma HS^b^ Center
8 Mountain	AT Still University of HS^b^	University of New Mexico-Main Campus	Northern Arizona University	Touro University Nevada	University of Utah	Midwestern University-Glendale			
9 Pacific	University of Southern California	Loma Linda University	Western University of HS^b^	Samuel Merritt University	University of Washington Seattle Campus	Touro University California			

*Abbreviations*: ^a^*MA* Massachusetts, ^b^*HS* Health Sciences

^c^(PA programs within each division are not rank ordered. The complete list and rankings of all top performing programs is available as a Supplemental Appendix 1 and 2. The rankings are based on both number of graduates [Supplemental Appendix 1] and proportion of graduates [Supplemental Appendix 2] of PA graduates per division.)

### Total number of graduates per program

**Fig. 2:**
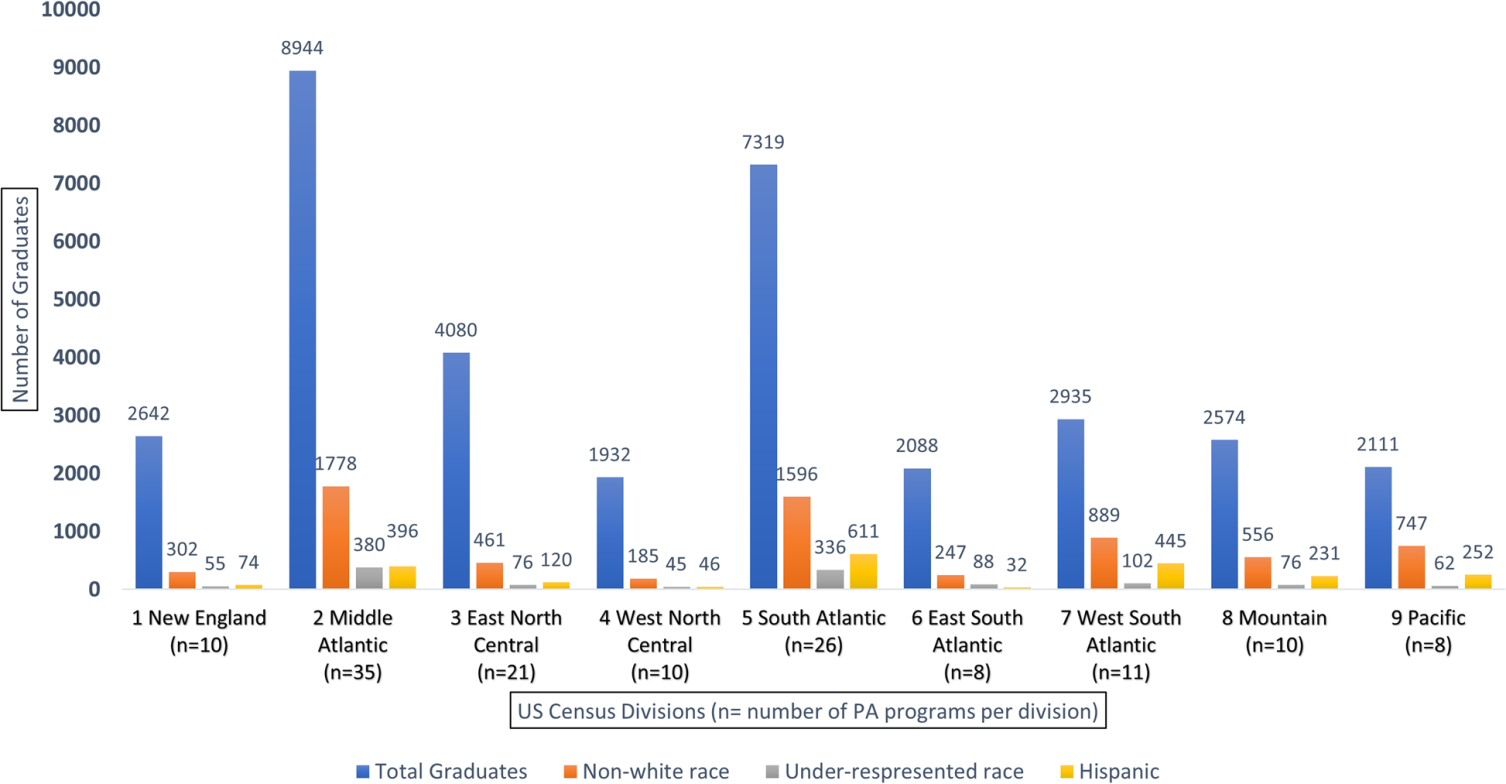
Number of PA graduates per US Census Divisions from 2014–2018 (*n* = 139 programs)

Figure 2 displays the total number of graduates by category. Across all programs over 5 years, there were a total of 34,625 graduates. The number of graduates per Division ranged from a minimum of 1,932 (Division 4) to a maximum of 8,944 (Division 2) with a median of 2,642 graduates (Division 1). In comparison to the total number of white graduates, the total number of non-white graduates were significantly lower (chi-squared test for homogeneity *p*-value < 0.001). Only 6,761 out of the 34,625 (19.5%) graduates were of non-white over a five-year period. Furthermore, the number of non-white graduates was less than half of the total number of white graduates in any Division. Division 9 and Division 7 were the most diverse U.S. Divisions with 747 out of 2,111 (35.4%) and 889 out of 2,935 (30.3%) of their graduates classified as diverse PA graduates. Five out of 9 Divisions had less than 20% non-white race/ethnicity graduates. Across the U.S., proportions of Hispanics and URM race PA graduates were low. Only 2,207 (6.4%) were Hispanic, and 1,220 (3.5%) were of URM race.

Chi-squared tests for homogeneity between top performing programs versus all programs within each Division were highly statistically significant (*p*-values < 0.001) for all three categories (i.e., non-white race/ethnicity, URM race and Hispanic ethnicity). As a sensitivity analysis, the same analyses were conducted between top performing programs versus all other programs excluding top performers within each Division, but the results remained the same.

**Fig. 3:**
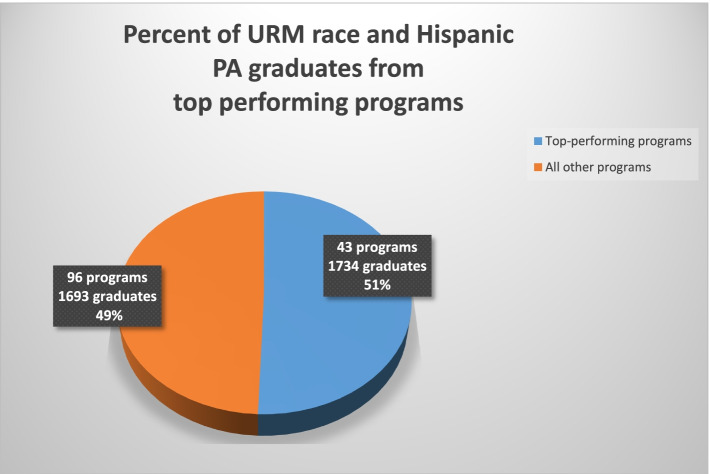
Percent of URM race and Hispanic PA graduates from top-performing programs

**Fig. 4:**
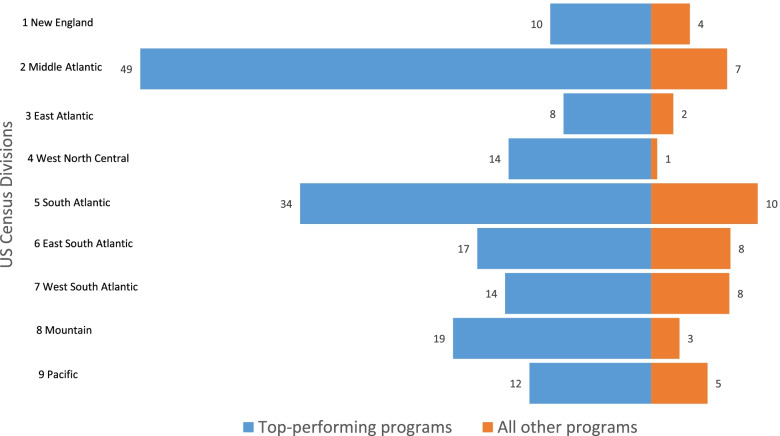
Average number of URM race PA graduates per program per U.S. Census Division over 5 years (2014–2018)

**Fig. 5:**
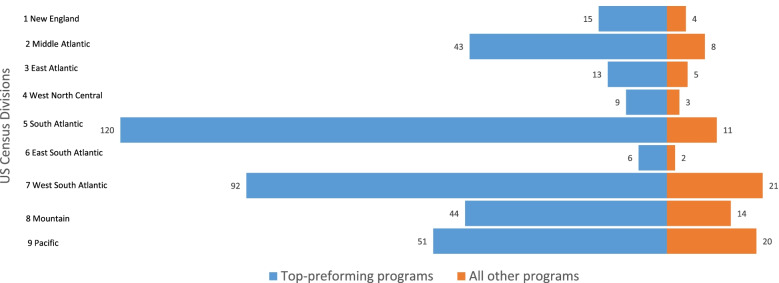
Average number of Hispanic PA graduates per program per U.S. Census Division over 5 years (2014–2018)

A substantial number of Hispanic and URM race graduates came from a few top performing programs. Comparing top performing programs with all programs, Fig. 3 shows that during the study period, over half of the URM and Hispanic graduates (51%) came from under a third of all programs (31%) classified as top performing. This trend is similarly reflected within each Division. Figures 4 and 5 show the average number of URM and Hispanic graduates per program over the study period between top performing programs versus all others in each Division. Figures 4 and 5 show the average number of graduates rather than raw totals because different Divisions have different numbers of programs. For details of counts of graduates per program, refer to the Supplemental Appendix Top Performers Proportions and Number of Graduates.

### Top performers with low diversity

Some programs were identified as top performers by being in a division with overall low diversity. For example, in Division 6, three programs were identified as top performers in Hispanic graduates despite having only 5 Hispanic graduates in 5 years. Similarly, in Division 4, the second top performer for URM race only had 5 URM race graduates in 5 years. Lastly in Division 4, three programs tied for third place in the URM race category with only 3 graduates each.

### Proportion of graduates per program

Table 2 displays the average proportions of graduates by diversity category per Division. There was a wide range in diversity proportions between programs within most Divisions. For example, in Division 2, although the average proportion of non-white graduates was 20.3%, the proportion of graduates who were of non-white ranged from 1.4% to 86.2%. In Division 2, the difference in average proportions of non-white race between all programs in the division and top performing programs was 20.3% vs. 70.2%, respectively. There were some Divisions where the range was much narrower, but these were Divisions where the proportions were generally low. For example, in Division 6, the proportion of Hispanic graduates ranged from 0.4–3.4%. Across all programs, the proportions of URM race graduates were very low, ranging from 1.8% (Division 4) to 4.7% (Division 2).

**Table 2:** Proportion of diverse PA graduates: All PA programs per division (*n* = 139) compared to the top 3 performing PA programs per division

Division *(n* = *number of PA programs)*	Non-white race		URM race		Hispanic	
	All PA programs	Top 3 performing PA programs	All PA programs	Top 3 performing PA programs	All PA programs	Top 3 performing PA Programs
**1 New England (*n* = 10)**	12.0%	16.3%	2.4%	4.2%	3.1%	5.3%
**2 Middle Atlantic (*n* = 35)**	20.3%	70.2%	4.7%	24.4%	4.8%	20.9%
**3 East North Central (*n* = 21)**	10.9%	20.3%	1.9%	7.2%	2.8%	5.6%
**4 West North Central (*n* = 10)**	9.0%	14.0%	1.8%	4.1%	2.3%	3.6%
**5 South Atlantic (*n* = 26)**	20.7%	50.1%	4.7%	10.5%	7.3%	29.7%
**6 East South Atlantic (*n* = 8)**	11.7%	17.9%	4.4%	7.0%	1.6%	2.7%
**7 West South Atlantic (*n* = 11)**	28.7%	45.8%	3.8%	5.5%	14.2%	31.1%
**8 Mountain (*n* = 10)**	22.7%	36.9%	2.5%	5.1%	10.8%	22.7%
**9 Pacific (*n* = 8)**	35.1%	49.1%	3.0%	4.2%	12.0%	16.9%

### Proportion of graduates compared to the U.S. census general population per division

Table 3 displays the proportion of PA graduates compared to U.S. Census General Population of racial and ethnic demographics per Division. In each Division, the proportions of white and non-white PA graduates were comparable to the general U.S. population. However, URM race and Hispanic PA graduates were disproportionately lower.

**Table 3:** PA graduate demographics per U.S. Census Division compared to general population demographics per U.S. Census Division

**Division**	Count	White (%)	Non-white race (%)	URM race (%)	Hispanic (%)
1. New England					
General Population	14,845,063	80.2%	12.5%	7.5%	11.6%
PA Graduates	2,642	60.5%	11.4%	2.1%	2.8%
2. Mid Atlantic					
General Population	41,137,740	69.2%	21.6%	14.3%	16.1%
PA Graduates	8,944	71.2%	19.9%	4.2%	4.4%
3. East North Central					
General Population	46,902,431	78.6%	15.9%	12.4%	8.8%
PA Graduates	4,080	85.3%	11.3%	1.9%	2.9%
4. West North Central					
General Population	21,426,573	83.9%	11.3%	8.3%	6.6%
PA Graduates	1,932	82.6%	9.6%	2.3%	2.4%
5. South Atlantic					
General Population	65,784,817	67.4%	26.5%	22.7%	14.7%
PA Graduates	7,319	69.7%	21.8%	4.6%	8.3%
6. East South Central					
General Population	19,176,181	74%	22.5%	21%	4.5%
PA Graduates	2,088	72.0%	11.8%	4.2%	1.5%
7. West South Central					
General Population	40,619,450	72.2%	19.7%	15.6%	30.6%
PA Graduates	2,935	64.2%	30.3%	3.5%	15.2%
8. Mountain					
General Population	24,854,998	80.2%	10.8%	7.4%	25.3%
PA Graduates	2,574	74.4%	21.6%	3.0%	9.0%
9. Pacific					
General Population	53,492,270	62.2%	20.6%	6.9%	32.4%
PA Graduates	2,111	61.9%	35.4%	2.9%	11.9%

## Discussion

Despite immense growth in the PA profession over recent decades, the number of non-white graduates has remained disproportionately low. Utilizing data from IPEDS, the current study illustrates the results from the quantitative analysis. The goal of the quantitative study was to identify top performing PA programs per U.S. Census Division that have contributed to racially and ethnically diverse graduates from 2014–2018. Our analysis revealed several important findings. First, the total number of non-white graduates was far less than the total number of graduates across all Divisions (Fig. 2). Second, a significant number of URM and Hispanic graduates came from a few top performing programs (Figs. 3,4,5), and thus, without these top performing programs, the overall diversity numbers would be dismal. Third, similar trends of top performing programs accounting for the majority of diverse PA graduates were observed in the proportions of non-white graduates, irrespective of the size of the program (Table 2). Fourth, the proportions of URM race and Hispanic PA graduates were significantly lower than the proportions of URM race and Hispanic residents observed in the corresponding U.S. Census Division (Table 3).

This study provides empirical evidence that the PA profession lags behind recommendations from our professional and accrediting organizations to increase workforce diversity of health professionals. Of 34,625 total graduates from 2014 to 2018, only 3,427 (9.9%) were either URM race or Hispanic. It is projected that the U.S. population will become “majority–minority” by the year 2044, and nearly one in five of the nation’s total population is projected to be foreign-born by 2060 [38]. This anticipated demographic shift could benefit from an increase in diverse providers capable of managing the unique challenges related to race and ethnicity. Health care providers identifying with an URM race or Hispanic ethnicity group are more likely to work in underserved and minority communities than those who do not identify as such [39–41]. The Institute of Medicine Unequal Treatment and the Sullivan Commission report provided evidence and recommendations for greater commitment to diversifying the health professions workforce to enhance health equity and reduce health disparities of the nation’s most vulnerable and marginalized populations [40]. Thus, there is an urgent need to innovate and develop interventions to solve the long-standing disparities in patient-provider concordance and to create a diverse PA workforce [42].

The PAEA supports and promotes holistic admissions practices to increase the diversity of PA matriculants. However, the findings from this study indicate that only a small number of PAEA-affiliated programs are contributing diversity to the PA workforce. Of the 1,220 URM race graduates, 544 came from only 29 top performing programs out of the 139 programs in the study. Similarly, 44.6% of all URM race graduates came from only 20.9% of all programs. Likewise, 53.9% of all Hispanic graduates came from 20.1% of programs. To ensure that these differences were not simply due to the size of the programs, we also considered the proportion of diverse graduates per program. These analyses showed similar results, where the top performing programs had much higher average proportions of URM race and Hispanic ethnicity graduates.

Additionally, to ensure that differences were not due to geographic differences in racial/ethnic makeup of various parts of the U.S., we compared our findings with the general U.S. population in each Division. These comparisons allow us to account for the demographic differences that may occur across the country. Each Division is composed of several neighboring states, which are more likely to have racial and ethnic similarity. In general, students are more likely to apply to PA programs located within a division inclusive of the states surrounding their home state rather than to apply to PA programs at a national level. Moreover, many state institutions are required to admit a certain number or percentage of in-state applicants. Thus, PA programs are more likely to compete for students within their division. The proportions of URM race and Hispanic PA graduates were significantly lower compared to the general population in every Division.

## Limitations

In addition to the illumination of the racial and ethnic disparities in PA graduates, another major strength of the current study is the use of individual program level data to explore diversity. The percentage of racial and ethnic diverse first-year PA student matriculants and the number of certified PAs have historically been reported in aggregate for the profession. To date, there have been no published studies revealing individual PA program racial and ethnic graduation trends. Another strength of the current study is that while it is discouraging that many PA programs are not graduating more URM race and Hispanic students, our findings justify the need for our ongoing qualitative follow-up study to determine the reasons why some programs are doing better than others. Lastly, the methodology used in the current study can be extended to investigate diversity of any health profession education program represented in the IPEDS database, e.g., medicine, pharmacy, dentistry, nursing, physical therapy, etc.

Despite the many strengths, this study is not without its limitations. First, five institutions with affiliated programs reported data in aggregate of multiple programs under one institutional name representing a total of 12 individual PA programs. However, as we had 139 programs in the study sample, we do not believe that disaggregating these programs would have made a significant difference to our results. Second, there is a general assumption that all graduates become practitioners, and that the diversity of the educational pipeline will reflect the diversity of new practitioners. While some PA graduates may not practice clinically in their future professions, it is safe to assume that the number of diverse graduates strongly correlates with the number of diverse practitioners. Furthermore, to our knowledge, there is no evidence which indicates that certain racial or ethnic groups enter clinical practice at lower rates than other groups. Colleges and Universities that receive Title IV funding submit data annually to IPEDS. Comparing top performers to U.S. Census Division populations could be viewed as a shortcoming and opportunity for further research. However, the comparison allows for consistency with historical data reporting by PAEA, the only association in the U.S. that oversees PA education. A final potential limitation of the current study, it is possible that user or administrative errors occurred when submitting data on PA graduates across hundreds of categories [30]. We believe that the potential for these classification errors is low and such an error should be equivalently distributed across programs. Lastly, some programs were chosen as “top performers” despite having a very low number of diverse graduates due to being in a division with overall low diversity. However, there was no way of knowing a priori that this would be the case when we decided the selection criteria for this study.

## Conclusions

Despite the abundance of evidence for the need to diversify the healthcare workforce, PA programs have had difficulty recruiting and graduating a diverse group of students. This study provides empirical evidence that PA programs have not been able to attain the level of diversity necessary to shift the lack of diversity in the PA workforce. Next steps will consist of the analysis of qualitative data from top performing programs in order to establish PA diversity benchmarks and best practices that are reproducible across the country.

## Supplementary Information

**Additional file 1: Supplemental Appendix 1.** Top PA Performers Ranked by Number of Graduates from 2014-2018. 

**Additional file 2: Supplemental Appendix 2.** Top PA Performers Ranked by Proportion of Graduates from 2014-2018.

## Data Availability

Physician Assistant graduate data were retrieved from the Integrated Postsecondary Education Data System, obtained from annual surveys conducted by the U.S. Department of Education’s National Center for Education Statistics. The dataset supporting the conclusions of this article can be retrieved from the National Center for Education Statistics website https://nces.ed.gov/ipeds/use-the-data. Datasets used and/or analyzed during the current study are available from the corresponding author on reasonable request.

## Abbreviations

AAPA: American Academy of Physician Assistants
ARC-PA: Accreditation Review Commission on Education for the Physician Assistant
CASPA: Central Application Service for Physician Assistants
CIP: Classification of Instructional Programs
IPEDS: Integrated Postsecondary Education Data System
NCCPA: The National Commission on Certification of Physician Assistants
PA: Physician Assistants
PAEA: Physician Assistant Education Association
PANCE: Physician Assistant National Certifying Examination
URM: Underrepresented Minority
US: United States
